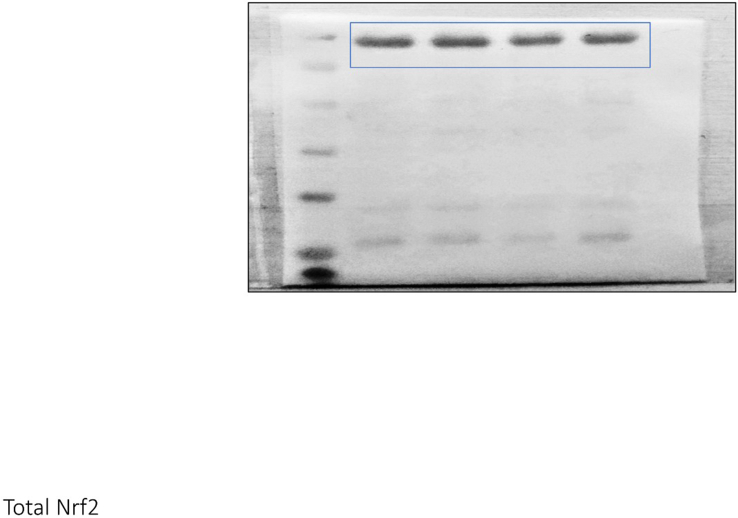# Berberine alleviates chlorpyrifos-induced nephrotoxicity in rats via modulation of Nrf2/HO-1 axis

**DOI:** 10.1016/j.heliyon.2024.e25233

**Published:** 2024-01-29

**Authors:** Lenah S. Binmahfouz, Emad H.M. Hassanein, Amina M. Bagher, Rawan H. Hareeri, Zaenah Z. Alamri, Mardi M. Algandaby, Mohamed M. Abdel-Daim, Ashraf B. Abdel-Naim

**Affiliations:** a Department of Pharmacology and Toxicology, Faculty of Pharmacy, King Abdulaziz University, Jeddah, 21589, Saudi Arabia; b Department of Pharmacology and Toxicology, Faculty of Pharmacy, Al-Azhar University, Assiut, Egypt; c Department of Biological Sciences, College of Science, University of Jeddah, Jeddah, Saudi Arabia; d Department of Biological Sciences, Faculty of Science, King Abdulaziz University, Jeddah, 21589, Saudi Arabia; e Medicinal Plants Research Group, Deanship of Scientific Research, King Abdulaziz University, Jeddah, 21589, Saudi Arabia; f Department of Pharmaceutical Sciences, Pharmacy Program, Batterjee Medical College, Jeddah, 21442, Saudi Arabia; g Pharmacology Department, Faculty of Veterinary Medicine, Suez Canal University, Ismailia, 41522, Egypt

**Keywords:** Chlorpyrifos, Berberine, Nephrotoxicity, Keap1/Nrf2/HO-1, NF-κB

## Abstract

Chlorpyrifos (CPS), an organophosphorus insecticide, is widely used for agricultural and non-agricultural purposes with hazardous health effects. Berberine (BBR) is a traditional Chinese medicine and a phytochemical with anti-inflammatory and anti-oxidative properties. The present study evaluated the effects of BBR against kidney damage induced by CPS and the underlying mechanisms. An initial study indicated that BBR 50 mg/kg was optimal under our experimental conditions. Then, 24 rats (6/group) were randomized into: control, BBR (50 mg/kg/day), CPS (10 mg/kg/day), and CPS + BBR. BBR was administration 1 h prior to CPS. Each treatment was delivered daily for a period of 28 consecutive days using a gastric gavage tube. Compared to CPS-alone treated rats, BBR effectively improved renal function by preventing the rise in serum urea, creatinine, and uric levels. The reno-protective effects of BBR were confirmed through a histological examination of kidney tissues. BBR restored oxidant-antioxidant balance in renal tissues mediated by Keap1/Nrf2/HO-1 axis modulation. In addition, BBR decreased nitric oxide (NO) and myeloperoxidase (MPO) activity. This was paralleled with the potent down-regulation of NF-κB. Furthermore, BBR exhibited anti-apoptotic activities supported by the upregulation of Bcl-2 and down-regulation of Bax and caspase-3 expression. In conclusion, our data suggest that BBR attenuates CPS-induced nephrotoxicity in rats by restoring oxidant-antioxidant balance and inhibiting inflammatory response and apoptosis in renal tissue. This is mediated, at least partly, by modulation of the Nrf2/HO-1 axis.

## 1 Introduction

Chlorpyrifos (CPS), an organophosphorus insecticide, is widely used for agricultural and non-agricultural purposes with definite hazardous health effects [1]. CPS can be ingested, inhaled, and absorbed via the skin during preparation and application and through consuming contaminated foods [2]. The adverse impacts of organophosphates on renal tissues and kidney function have been reviewed and substantiated [3]. Experimentally, CPS nephrotoxicity has been well-documented [[4], [5], [6]]. In addition to cholinesterase inhibition, CPS toxicity has been shown to include inflammation, oxidative and nitrosative stress, and apoptosis, eventually leading to cellular damage [[7], [8], [9]].

Nuclear factor-κB (NF-κB), a regulator of inflammation, controls the expression of pro-inflammatory genes in several cell types, including the kidney [10]. Further, the primary defense mechanism against oxidative and electrophilic stressors is the Kelch-like ECH-associated protein 1 (Keap1)/nuclear factor erythroid 2–related factor 2(Nrf2)/heme oxygenase-1 (HO-1) pathway [11]. One of the downstream antioxidants is HO-1, which also inhibits inflammatory responses by catalyzing the breakdown of heme [12,13]. Additionally, numerous studies indicated that repeated exposure to CPS induces the production of pro-apoptotic proteins like Bcl-2-associated X protein (Bax) and inhibition of anti-apoptotic proteins like Bcl-2. One of the most significant proteins released during the intrinsic and extrinsic apoptotic pathways is the executioner caspase 3 [[14], [15], [16]]. Thus, anti-apoptotic agents are expected to be beneficial in treating renal intoxication induced by CPS.

Numerous studies indicated that various phytochemicals have therapeutic potential for treating multiple ailments [17,18]. Additionally, considerable studies reported that phytochemicals effectively attenuate CPS-associated toxicities [19,20]. Berberine (BBR) is a phytochemical of traditional medicinal uses and has promising effectiveness in treating several renal problems [21]. BBR has a wide range of biological activities [22]. Notably, several studies revealed that BBR could reduce oxidative stress by modulating the Keap1/Nrf2 signaling pathway [[23], [24], [25]]. Moreover, BBR inhibits inflammatory cytokines and apoptosis, which suggests that it can mitigate kidney damage by reducing the generation of inflammatory mediators and enhancing Bcl-2 expression [[25], [26], [27]]. In this regard, BBR showed protective benefits against various chemicals' toxicity. In particular, BBR proved effective against experimentally-induced kidney injury [[28], [29], [30]]. However, its efficacy against CPS-induced renal intoxication has not yet been studied. Therefore, this study aimed to investigate the protective effects of BBR against kidney damage induced by CPS and the key molecular mechanisms implicating the Keap1/Nrf2/HO-1 axis and NF-κB signaling in these effects.

## 2 Materials and methods

### 2.1 Chemicals

CPS was obtained from the Egyptian Pesticides and Chemicals Company (EPIC), Cairo, Egypt. BBR was purchased from Xi'an Saiyang Bio-technology (Shaanxi, China). CPS and BBR were suspended in 1 % carboxymethyl cellulose (CMC). All other chemicals were of the highest purity.

### 2.2 Animals

A total of 54 12-weeks-old male Wistar rats (200 ± 20 g) were purchased from the Faculty of Pharmacy, King Abdulaziz University, Jeddah. The animals were acclimated to 22 ± 2 °C and kept on a 12-h light/dark cycle, with unlimited access to water and food for at least one week before the experiment. Thirty rats were used in a pilot experiment to determine the optimal dose of BBR. Then, the chosen dose was used in a subsequent full experiment. All animal procedures were approved by the Research Ethics Committee, King Abdulaziz University (Ref # PH-1443-69). In all investigations, the National Institute of Health guidelines for the care and use of laboratory animals (NIH Publications No. 8023, revised 1978) were followed.

### 2.3 Pilot study

A pilot study was first conducted to assess the efficacy of BBB in our study. Thirty male rats were randomly divided into five groups (6/group). The following groups were assigned: Group I acted as the control group (1 % CMC), whereas Group II received CPS at a dose of 10 mg/kg, based on previously published data [31], Group III received 10 mg/kg CPS in combination with 25 mg/kg of BBB, Group IV received 10 mg/kg CPS in combination with 50 mg/kg of BBB, and Group V received 10 mg/kg CPS in combination with 100 mg/kg of BBB. BBR was administered 1 h before CPS. All treatments were administred orally daily for 28 consecutive days using a gastric gavage tube at a dosing volume of 10 ml/kg.

At 24 h following the last treatment, the animals were anesthetized with an IP injection of ketamine (80 mg/kg). Blood was then drawn from the retro-orbital plexus. The collected blood samples were allowed to clot for 15 min at room temperature, followed by centrifugation at 3000 rpm for 15 min to separate the sera. Serum urea, creatinine, and uric acid levels were examined as indications of BBB efficacy. Based on the findings of this pilot study, a dose of 50 mg/kg of BBB was used for the following experiments.

### 2.4 Experimental design

Herein, male 24 rats were randomly divided into four groups (6/group). Group I (Control 1 % CMC alone), Group II (BBR 50 mg/kg), Group III (CPS 10 mg/kg), and Group IV (BBR 50 mg/kg + CPS 10 mg/kg) at doses of 50 mg/kg and 10 mg/kg, respectively. In groups III and IV, BBR was given 1 h before CPS. All treatments were given once daily using a gastric gavage tube and continued for 28 successive days.

At 24 h after the last treatment, animals were anesthetized using IP ketamine (80 mg/kg), and blood samples were collected from the retro-orbital plexus. The blood samples were left for 15 min at room temperature to coagulate and then centrifuged at 3000 rpm for 15 min to obtain sera. The animals were sacrificed by decapitation, abdomens were opened, and kidneys were rapidly collected. Representative kidneys were kept in 10 % neutral buffered formalin for histopathology and immunohistochemistry studies. The remaining kidneys were flash-frozen in liquid nitrogen and stored at −80 °C with sera for subsequent biochemical analyses.

### 2.5 Kidney function biomarkers

The levels of creatinine, urea, and uric acid in serum were determined using commercial vendor kits with the catalogue numbers CR-12-50, UR-21-10, and UA-21-20, respectively (Diagnostic, Giza, Egypt). Serum cystatin C levels were determined using kit number MSCTC0 (R&D Systems Inc., Minneapolis, MN, USA) and neutrophil gelatinase-associated lipocalin (NGAL) levels were determined using kit number MBS260195 (Mybiosource, San Diego, CA, USA).

### 2.6 Histopathological assessments

Kidney tissue samples were formalin-fixed and paraffin-embedded with a thickness of 5-μm. The kidney sections were stained with H&E for light microscopy. For each group, a blinded pathological assessment of the histopathological changes was performed [32]. The degree of pathological changes was given a score of – (not detected), + (mild), ++ (moderate), or +++ (severe). Percentage of histopathological changes was estimated and statistically evaluated by non-parametric tests.

### 2.7 Assessment of renal oxidative stress biomarkers

Evaluation of renal malondialdehyde (MDA), reduced glutathione (GSH), and total nitrites (as a marker of NO) contents in the kidney hemogenates were assessed using commercial kits with catalog numbers MD-25-29, GR-25-11 and NO-25-33, respectively. Oxidized glutathione (GSSG) concentration was measured using kit number MBS752665 (Mybiosource, San Diego, CA, USA). Renal enzymatic activities of glutathione-S-transferase (GST), superoxide dismutase (SOD), and myeloperoxidase (MPO) were evaluated using commercial kits with numbers GT-25-19, SD-25-21, and MP-26-11 respectively. All kits were obtained from Diagnostic, Giza, Egypt.

### 2.8 Western blot

Kidney lysates were prepared on ice, then centrifuged for 10 min (14,000 rpm, at 4 °C). Lysate protein concentration was determined using a Protein Assay Kit I (Catalog # 5000006, Bio-Rad, Hercules, CA, USA). Then, lysate samples (50 μg protein) were separated by a 10 % Tris-Glycine gel and transferred for 2 h using a semi-dry transfer cell to a PVDF membrane (ab133411, ABCAM, Cambridge, UK). After separation, membranes were blocked with 5 % non-fat dry milk in TBST (Tris 0.01 M pH 7.4, 100 mM NaCl and 0.1 % Tween 20) for 30 min. This was followed by incubation overnight at 4 °C with one of the following primary antibodies: anti-Nrf2 (MBS714561), anti-Keap1 (MBS714561), anti–HO–1 (MBS220919) (MyBioSource, San Diego, CA, USA). Membranes were washed with TBST and incubated with HRP-conjugated secondary antibody for 1 h. Immunoreactivity was visualized using an enhanced chemiluminescence kit (GE Healthcare, Piscataway, NJ, USA). β-Actin was the reference protein using a primary antibody with catalog # ab8226 (ABCAM, Cambridge, UK).

### 2.9 Immunohistochemical examination

Bax, Bcl-2, caspase-3, and NF-κB were immunoassayed in kidney tissue sections of 5-μm-thickness [33]. Endogenous peroxidase activity was inhibited for 30 min by incubating with 0.3 % H_2_O_2_ in methanol. Microwave treatment (15 min) in sodium citrate buffer was used to retrieve antigens (0.1 M, pH 6.0). Tissue sections were then incubated overnight at 4 °C with rabbit anti-Bax anti-Bcl-2, anti-caspase-3, or NF-κB (p105/p50) for 30 min (Catalog # ab32503, ab196495, ab184787 or ab32360, respectively, ABCAM, Cambridge, UK). After several washings, the sections were incubated for 30 min with secondary antibodies. After that, the reaction was visualized using DAB as a chromogen. The area percent of brown color was calculated in six fields per slide using ImageJ software (ImageJ, 1.46a, NIH, Bethesda, MD, USA).

### 2.10 Statistical analysis

Results are expressed as mean ± SD. For the majority of results, a one-way ANOVA was utilized, followed by Tukey post-analysis test to determine the significance of difference between groups. For the evaluation of histopathological scoring, the Kruskal Wallis test was used followed by Dunn's multiple comparisons test. The analysis was conducted using GraphPad Prism® version 8. P < 0.05 was considered as the criterion of significance.

## 3 Results

### 3.1 Dose-response study of BBR on kidney function of CPS-treated rats

**Fig. 1:**
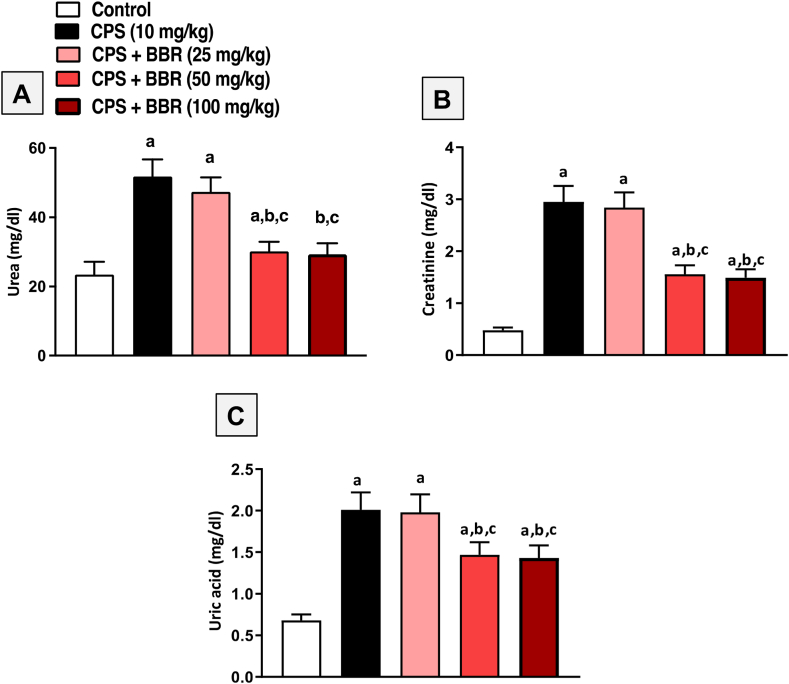
**Effect of different dose levels of BBR on markers of kidney function in CPS-treated rats. A**: Urea, **B**: Creatinine, **C**: Uric acid. Data are mean ± SD (n = 6). a, b, c significantly different from Control, BBR, or CPS, respectively, at *p* < 0.05 using one-way ANOVA followed by Tukey post-analysis.

Initially, a pilot study was carried out to discover the optimal dose of BBR for its possible therapeutic effects. Three dose levels of BBR (25, 50, and 100 mg/kg) were tested against CPS-induced nephrotoxicity, and serum urea, creatinine, and uric acid levels were measured. Fig. 1 shows that 10 mg/kg CPS treatment severely reduced renal function, reported by significantly increased levels of serum urea (Fig. 1A), creatinine (Fig. 1B), and uric acid (Fig. 1C). Co-treatment with BBR, on the other hand, enhanced kidney function in a dose-dependent manner. The optimal BBR dose was 50 mg/kg, which demonstrated a statistically significant protective effect compared to the lower dose of 25 mg/kg. The higher BBR dose (100 mg/kg) had no additional impact on kidney function compared to the optimal dose. As a result, the middle BBR dose (50 mg/kg) was chosen for subsequent experiments.

### 3.2 BBR attenuated kidney impairment in CPS-treated rats

**Fig. 2:**
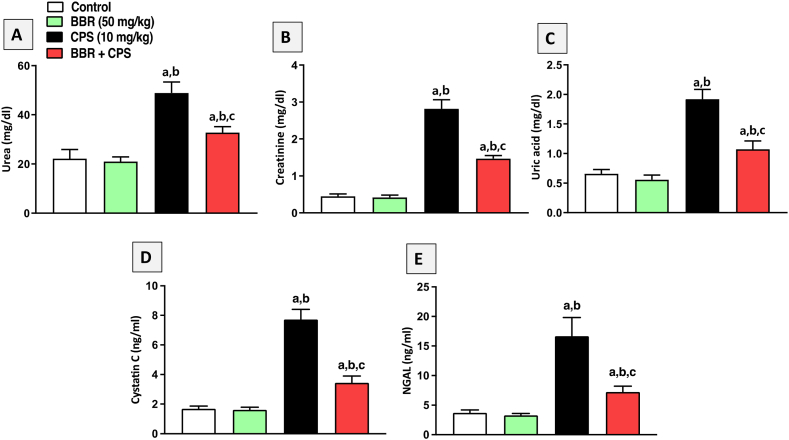
**Effect of BBR (50 mg/kg) on serum markers of kidney function in CPS-treated rats. A**: Urea, **B**: Creatinine, **C**: Uric acid, **D:** Cystatin C, **E:** NGAL. Data are mean ± SD (n = 6). a, b, c significantly different from Control, BBR, or CPS, respectively, at *p* < 0.05 using one-way ANOVA followed by Tukey post-analysis.

To explore the renoprotective effects of BBR against CPS-induced renal intoxication, serum urea (Fig. 2A), creatinine (Fig. 2B), uric acid (Fig. 2C), cystatin C (Fig. 2D), and NGAL (Fig. 2E) were assessed as renal function markers. As shown in Fig. 2, CPS (10 mg/kg) administration significantly increased the level of all tested markers compared to control rats. Co-treatment with BBR (50 mg/kg) significantly reduced CPS-induced kidney damage, as demonstrated by lower serum urea, creatinine, uric acid, cystatin C and NGAL levels by 33 %, 48 %, 44 %, 55 % and 56 % respectively, compared to the CPS-alone group.

### 3.3 BBR inhibited kidney histopathological changes in CPS-treated rats

**Fig. 3:**
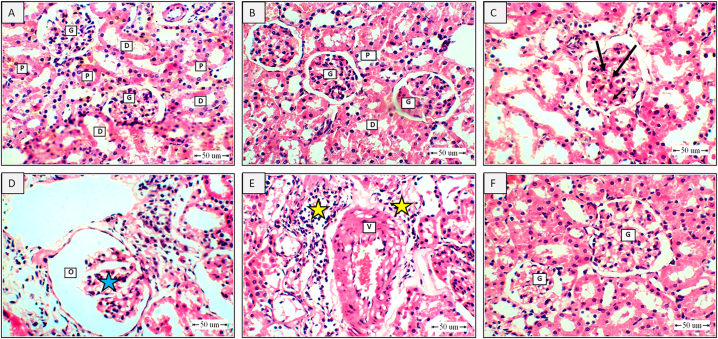
**Impact of BBR on kidney histopathological changes in CPS-treated rats. A** represents a photomicrograph obtained from a kidney of a control rat showing glomeruli (G) made of tufts of capillaries lined with endothelium on the basement membrane, pericytes, and intramesengial cells as well as proximal (P) and distal convolutes tubules (D). Also, similar findings were observed in **B**, which represents a photomicrograph of a kidney of a rat in the BBR (50 mg/kg) group with normal glomeruli (G), proximal (P), and distal convolutes tubules (D). On the other hand, rat kidneys that received 10 mg/kg CPS only showed marked thickening of the basement membranes of the capillary tufts (black arrow), as represented in **C**. Also, a substantial decrease of cellularity (blue star) of the glomeruli and obliteration of the capsule (O) were observed in 10 mg/kg CPS-treated rats, as shown in **D**. Further, a marked thickening of the blood vessel (V) and perivascular inflammatory cellular reaction (yellow star) were also detected as shown in **E.** In contrast, co-treatment with 50 mg/kg BBR potently attenuated these histopathological changes, as evidenced by apparently healthy glomeruli (G), as shown in **F.** H&E. Scale bar = 50 μm. (For interpretation of the references to color in this figure legend, the reader is referred to the Web version of this article.)

Kidneys from control and BBR-control rats (Fig. 3A–B) showed normal glomeruli made of tufts of capillaries lined with endothelium on the basement membrane, pericytes, and intramesengial cells, as well as proximal and distal convolutes tubules. However, kidney sections obtained from 10 mg/kg CPS-intoxicated rats (Fig. 3C–D) exhibited marked thickening of the basement membranes of the capillary tufts, substantial decrease of glomerular cellularity, and obliteration of the capsule. Further, a marked thickening of the blood vessel and the perivascular inflammatory cellular reaction was observed (Fig. 3E). In contrast, co-treatment with 50 mg/kg BBR attenuated these histopathological changes, as evidenced by apparently healthy glomeruli (Fig. 3F). The histopathological changes were semi-quantified with regard to tubular dilatation, tubular necrosis, tubular cell swelling, glomerular injury, and perivascular inflammatory cellular infiltration (Table 1).

**Table 1:**
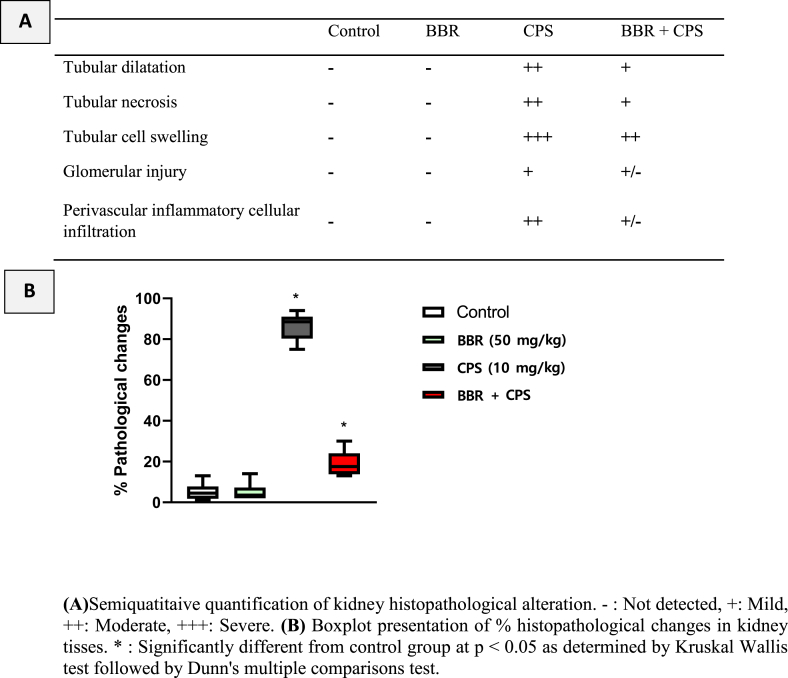
Effects of BBR on renal histopathology of CPS-treated rats.

### 3.4 BBR mitigated renal oxidative injury in CPS-treated rats

**Fig. 4:**
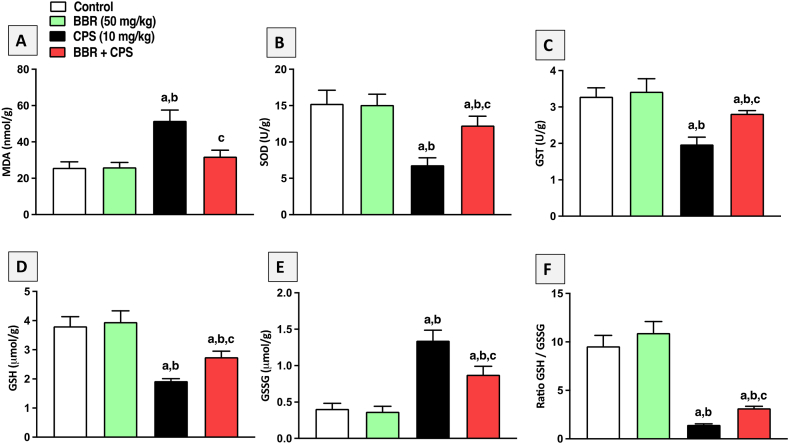
**Effect of BBR on renal oxidative injury in CPS-treated rats. A**: MDA, **B**: SOD, **C**: GST, **D**: GSH, **E:** GSSG, **F:** Ratio GSH/GSSG. Data are mean ± SD (n = 6). a, b, c significantly different from Control, BBR, or CPS, respectively, at *p* < 0.05 using one-way ANOVA followed by Tukey post-analysis.

The MDA (a marker of lipid peroxidation) and non-enzymatic (GSH and GSSG) and enzymatic (GST and SOD) antioxidants were assessed to evaluate the oxidative status in kidney tissues (Fig. 4). Challenging rats with CPS were associated with oxidative stress, as evidenced by a considerable increase in MDA content (Fig. 4A), exhaustion of SOD and GST activities (Fig. 4 B and C), a significant depletion of GSH concentration (Fig. 4D), and a substantial rise in GSSG concentration (Fig. 4E), as compared to the control group. However, BBR co-treatment significantly improved the oxidative status of kidney tissues. As compared to CPS-alone intoxicated animals, administration of 50 mg/kg BBR significantly prevented the rise in MDA content by 38 %, the decrease in GSH concentration by 42 %, the rise in GSSG by 34 %, and the decrease in GST and SOD enzymatic activities by 43 % and 80 %, respectively (Fig. 4A–F).

### 3.5 BBR upregulated protein expression of Keap-1, Nrf2 and HO-1 in kidneys of CPS-treated rats

**Fig. 5:**
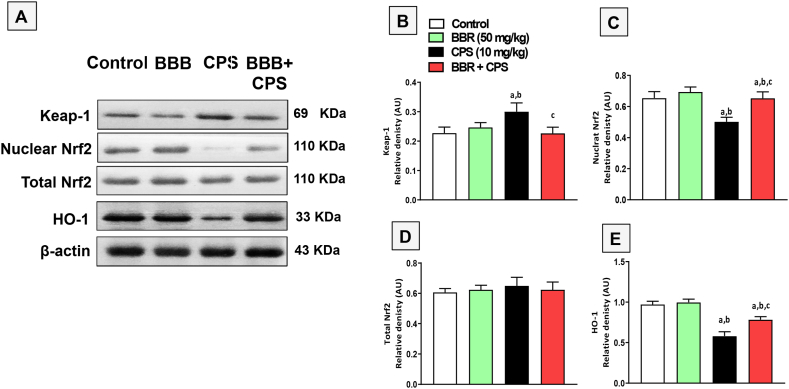
**Impact of BBR on kidney expression of Keap-1, nuclear Nrf2, total Nrf2, and HO-1 in kidneys of CPS-treated rats. A:** Western blot of control, BBR, CPS and BBR + CPS. **B**, **C**, **D**, and **E**: Graphical presentation of Keap-1, nuclear Nrf2, total Nrf2, and HO-1 relative optical densities, respectively. Data are mean ± SD (n = 6). a, b, c significantly different from Control, BBR, or CPS, respectively, at p < 0.05 using one-way ANOVA followed by Tukey post-analysis. Full, non-adjusted images of Fig. 5A are provided as Supplementary Material.

The effect of BBR on the expression of Keap-1, Nrf2, and HO-1 proteins in kidney tissues was investigated using Western blot analysis (Fig. 5A). Compared to the control groups, CPS significantly increased the expression of Keap-1 protein (Fig. 5B), decreased the expression of nuclear Nrf2 (Fig. 5C) with no significant changes in total Nrf2 expression (Fig. 5D) and inhibited HO-1 (Fig. 5E) in kidney tissues. However, compared to the CPS-only group, co-treatment with 50 mg/kg BBR significantly reduced Keap-1 by 24.4 % and enhanced nuclear Nrf2 and HO-1 expression by 46.9 % and 35.4 %, respectively.

### 3.6 BBR inhibited MPO activity and NO content in kidney tissues of CPS-treated rats

**Fig. 6:**
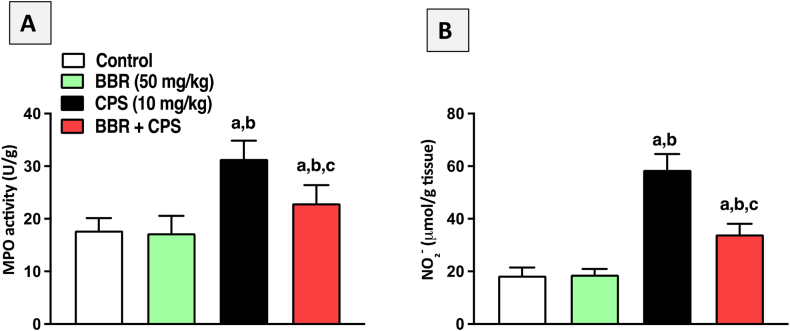
**Effect of BBR on MPO activity and total nitrite content in kidney tissues of CPS-intoxicated rats**. **A**: MPO, **B**: Total nitrite (NO_2_−). Data are mean ± SD (n = 6). a, b, c significantly different from Control, BBR, or CPS, respectively, at *p* < 0.05 using one-way ANOVA followed by Tukey post-analysis.

Following that, the effect of BBR on MPO activity and NO content in renal tissues of CPS-challenged rats was studied. Fig. 6 shows that CPS treatment significantly raised the neutrophil infiltration marker MPO activity (Fig. 6A) and total nitrite (NO_2_−) content (Fig. 6B) as a NO marker compared to the control groups. On the other hand, co-treatment with 50 mg/kg BBR significantly decreased MPO enzymatic activity by 27 % and NO_2_− concentration by 42 % in kidney tissues compared to CPS-alone treated rats.

### 3.7 BBR prevented the expression of NF-κB (p65) in kidney tissues of CPS-treated rats

**Fig. 7:**
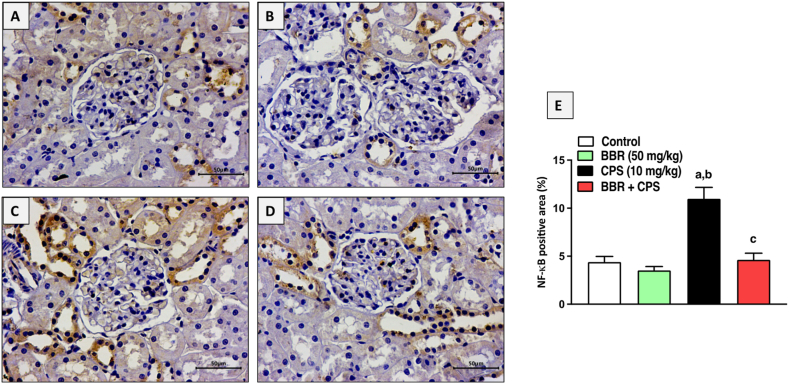
**Effect of BBR on NF-κB2 expression in kidney tissues of CPS-treated rats. A** Control, **B** BBR, **C** CPS, **D** BBR + CPS, and **E** graphic presentation of % positive areas in each section. Data are mean ± SD (n = 6). a, b, c significantly different from Control, BBR, or CPS, respectively, at *p* < 0.05 using one-way ANOVA followed by Tukey post-analysis.

Immunohistochemical assessment of nuclear factor-kappa B (NF-κB) expression in kidney tissues indicated that CPS administration resulted in significant up-regulation compared to control groups. Nevertheless, co-treating the rats with 50 mg/kg BBR significantly prevented the rise in NF-κB expression by 58 % compared to CPS-alone treated rats and successfully brought it to almost control values (Fig. 7A–E).

### 3.8 BBR attenuated apoptosis in CPS-treated rats

**Fig. 8:**
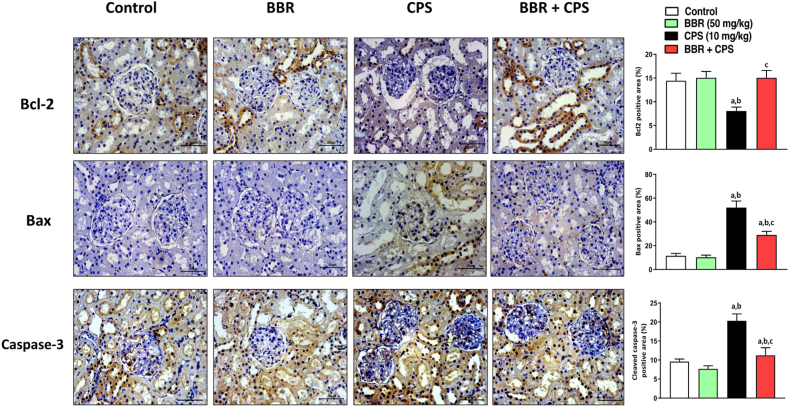
**Effect of BBR on the expression of Bcl-2 (upper panel), Bax (middle panel), and caspase-3 (lower panel) in kidney tissues of CPS-treated rats.** Bar charts represent semi-quantification of expression of corresponding proteins. Data are mean ± SD (n = 6). a, b, c significantly different from Control, BBR, or CPS, respectively, at *p* < 0.05 using one-way ANOVA followed by Tukey post-analysis.

As shown in Fig. 8, kidney sections from CPS-alone animals exhibited significant downregulation of Bcl-2 expression and upregulation of Bax and caspase-3 compared to control groups. However, the kidney sections of BBR co-treated rats showed a noticeable enhancement of Bcl-2 expression by 87 % (upper panel) and inhibition of Bax and caspase-3 expression by 44 % and 45 %, respectively (middle and lower panels, respectively) when compared to CPS-alone treated rats.

## 4 Discussion

CPS contaminates the environment by direct pesticide application through spray drift or foliar wash-off [34]. It poses health hazards, including nephrotoxicity [35]. Based on a pilot study and the data published in previous studies [31], CPS at a dose of 10 mg/kg of was used to induce nephrotoxicity in the current study. In this study, the potential of BBR is to combat CPS kidney toxicity in rats. Initially, a dose-response pilot study was conducted to establish the appropriate dose of BBR for potential therapeutic effects. Based on the results of the pilot study, both the 50 mg/kg and 100 mg/kg doses offered similar levels of protection, with the with 50 mg/kg showing a maximal response. Therefore, 50 mg/kg was used as the optimal dose for the subsequent experiments.

Serum levels of urea, creatinine, uric acid, cystatin C and NGAL were evaluated as renal function parameters to confirm the nephroprotective effects of BBR against CPS-induced renal intoxication. Recent research suggests that cystatin C may be a more accurate alternative to creatinine for estimating glomerular filtration rate (GFR) [36]. Unlike creatinine, cystatin C is not influenced by muscle mass, age, or sex, making it a potentially more reliable marker for estimating GFR. In addition, NGAL is produced by kidney tubule cells in response to kidney injury [37]. Its levels increase early after kidney damage, making it a valuable marker for detecting acute kidney injury [38]. Our data indicated that administering CPS caused a notable rise in the level of all tested markers compared to the control group. These findings are in harmony with several studies [7,39]. Contrarily, in rats administered oral BBR, these elevations were significantly prevented. This is in line with previous studies highlighting the nephroprotective characteristics of BBR in different experimental models [26,40,41]. These findings were confirmed by histological examinations of kidney tissues, indicating that rats exposed to CPS exhibited histopathological alterations in kidney tissues. This is supported by a previous report revealing that CPS nephrotoxicity was accompanied by hemorrhage, tubular necrosis, and mononuclear cell infiltration [6]. However, the results obtained in this study showed that BBR significantly alleviated such pathological changes in kidney tissues.

In general, oxidative renal damage is pivotal in organophosphate toxicity [42]. In particular, it is a key to CPS-induced nephrotoxicity pathogenesis [[7], [8], [9]]. According to an earlier study, CPS causes excessive free radicals production, which disables intracellular antioxidant defense mechanisms and causes peroxidation of membrane polyunsaturated fatty acids [43]. The lipid peroxidation biomarker MDA and the antioxidants GSH and its ratio with GSSG, GST, and SOD were assessed to evaluate oxidative status in kidney tissues. CPS was found to cause oxidative stress, evidenced by increasing MDA content and lowering GSH concentration, GST, and SOD activities. Supplementing the rats with BBR greatly enhanced the antioxidant status by preventing the rise in MDA and improving GSH content and GST and SOD enzymatic activities. These data gain support from the previously reported antioxidant properties of BBR that afforded it the potential to combat many ailments [44], including kidney diseases [21].

One crucial aspect in understanding the mechanism of organophosphate toxicity, such as CPS, is the role of oxidative stress. It is well-established that exposure to organophosphates can lead to an imbalance between the production of reactive oxygen species (ROS) and the antioxidant defense system in various organs, including the kidneys [42]. The resulting oxidative stress can contribute to cellular damage and dysfunction. In this study, we focused on the Keap1/Nrf2/HO-1 axis, which has been widely recognized as the principal protective pathway against oxidative stress [11]. The Keap1/Nrf2/HO-1 axis plays an important role in maintaining cellular redox homeostasis by regulating the expression of antioxidant enzymes, detoxification enzymes, and various cytoprotective proteins. Activation of this pathway can enhance the cellular defense mechanisms against oxidative stress-induced damage. Therefore, the BBB-induced activation of the Keap1/Nrf2/HO-1 axis was further confirmed by western blotting.

Results from western blotting indicated that CPS significantly increased Keap1 protein expression while significantly decreasing Nrf2 nuclear translocation and HO-1 protein expression. According to several reports, Nrf2 activation effectively defends against the nephrotoxicity of different etiologies [45,46]. Oxidative stress activates Nrf2-dependent cytoprotective signaling, and HO-1 is one of the several cytoprotective genes whose transcription is stimulated by this nuclear translocation. HO-1 breaks down free hemoglobin to release free iron, carbon monoxide (CO), and biliverdin, which have anti-apoptotic, antiproliferative, and anti-inflammatory properties [47]. It is well known that CPS suppresses Nrf2 activation and lowers antioxidant levels in various organs and *in-vitro* studies [[48], [49], [50]]. Our findings demonstrated that BBR treatment reversed these effects and increased Nrf2 nuclear translocation and upregulated HO-1 protein while downregulated Keap1. Likewise, BBR demonstrated antioxidant action in various animals and cell lines [51]. These data implicate that Keap1/Nrf2/HO-1 axis is the underlying pathway for BBR's antioxidant activities.

The role of inflammation in organophosphates, particularly CPS-induced renal injury, has been previously reported [7,52]. This aligns with the observed oxidative stress in CPS-intoxicated rats in this study. Essentially, CPS's pro-inflammatory and pro-oxidant activities are strongly connected [53]. MPO is an enzyme that causes inflammation and oxidative stress by promoting the production of ROS and reactive nitrogen species (RNS) in infiltrating neutrophils [54]. Notably, levels of MPO enzymatic activity and NO content were elevated in CPS-challenged animals. Nevertheless, BBR administration significantly prevented the rise in MPO activity and NO content in kidney tissues. The observed anti-inflammatory activities of BBR align with several previous studies [55,56]. Importantly, NF-κB induces the expression of various pro-inflammatory genes, including those encoding chemokines and cytokines like IL-1β, TNF-α, and others [10,57]. In the current study, NF-κB was remarkably upregulated in CPS-challenged rats, while the administration of BBR significantly inhibited it. The impact of NF-κB suppression by BBR has been investigated in several investigations. In an earlier study, BBR exhibited renal anti-inflammatory effects in the alloxan model of diabetic rats via suppression of NF-κB activation [58].

Further, BBR was reported to reduce the production of pro-inflammatory cytokines and NF-κB activation in rat models of diabetic nephropathy [26]. Besides, BBR inhibited the NF-κB pathway and reduced lipopolysaccharide-induced inflammatory responses in mouse inner medullary collecting duct-3 cells [59]. Thus, our findings provide additional evidence for the ability of BBR to inhibit NF-κB signaling and guard against CPS-induced kidney injury.

Apoptosis has been reported to mediate CPS toxicity in different cell types [[60], [61], [62]]. The current study assessed Bcl-2, Bax, and caspase-3 protein expression in kidney tissues. Our findings displayed that CPS intoxication resulted in the enhancement of apoptotic el death. This was evidenced by decreased expression of Bcl-2 and increased expression of Bax and caspase-3 in the kidney tissues. These data are in line with several previous studies [7,9]. In the current investigation, the administration of BBR resulted in enhanced expression of Bcl-2 and reduced expression of Bax and caspase 3. Therefore, targeting the apoptotic pathway is key to BBR renal protective effects. These are in line with previous studies [25,63].

One limitation of our study is the relatively high effective dose of BBB used in our experimental design. The use of a 50 mg/kg dose of BBB may raise concerns regarding the safety of implementing our approach. Therefore, new formulations are recommended to achieve comparable or improved outcomes with reduced dosage. By investigating alternative delivery systems, such as controlled-release mechanisms or targeted drug delivery, we can potentially optimize the therapeutic efficacy of BBB while minimizing the required dose. This would enhance the feasibility and applicability of our findings in future studies.

Several therapeutic approaches are clinically used for the management of organophosphorus-induced toxicity. The two main treatment choices are muscarinic receptor blockers and cholinesterase activators [64]. Muscarinic receptor blockers, such as atropine, are important in the treatment of organophosphorus insecticide poisoning. These agents competitively antagonize the muscarinic effects of excessive acetylcholine accumulation, which is one of the primary mechanisms underlying the toxic effects of organophosphorus compounds [65]. Pralidoxime, a cholinesterase activator, is another crucial component of the clinical treatment procedure. Organophosphorus compounds irreversibly inhibit acetylcholinesterase, resulting in acetylcholine accumulation and cholinergic toxicity. Pralidoxime can reactivate the inhibited acetylcholinesterase, restoring normal cholinergic function and decreasing the toxic effects of organophosphorus compounds [65]. Additionally, anti-inflammatory agents, such as interleukin-10 (IL-10), have showed promise in reducing the inflammatory response associated with organophosphorus poisoning [66]. Organophosphorus compounds have the ability to initiate an inflammatory cascade, resulting in the production of pro-inflammatory cytokines and the activation of inflammatory pathways. As an anti-inflammatory cytokine, IL-10 can modulate this response and help mitigate tissue damage. These interventions aim to reduce the toxicity of organophosphorus chemicals while also promoting renal repair.

## 5 Conclusion

Taken together, BBR has a protective effect against CPS-induced renal intoxication via its antioxidant, anti-inflammatory, and anti-apoptotic effects by regulating Keap1/Nrf2/HO-1 and apoptosis signaling pathways.

## Funding

The 10.13039/501100022230Deanship of Scientific Research (DSR) at 10.13039/501100004054King Abdulaziz University, Jeddah, Saudi Arabia, has funded this project, under grant no. (RG–86–130–42).

## Ethics declaration

All animal procedures were in accordance with the National Institutes of Health – Office of Laboratory Animal Welfare policies and laws. All animal studies complied with the ARRIVE guidelines and were approved by the Research Ethics Committee, King Abdulaziz University (Ref # PH-1443-69).

## Data availability statement

Data will be made available upon request.

## CRediT authorship contribution statement

**Lenah S. Binmahfouz:** Writing – review & editing, Writing – original draft, Methodology, Formal analysis. **Emad H.M. Hassanein:** Writing – original draft, Resources, Methodology, Formal analysis, Data curation. **Amina M. Bagher:** Resources, Methodology, Data curation. **Rawan H. Hareeri:** Resources, Methodology, Data curation. **Zaenah Z. Alamri:** Resources, Methodology, Formal analysis, Data curation. **Mardi M. Algandaby:** Writing – original draft, Resources, Methodology, Formal analysis, Data curation, Conceptualization. **Mohamed M. Abdel-Daim:** Writing – original draft, Resources, Methodology, Formal analysis, Data curation. **Ashraf B. Abdel-Naim:** Writing – review & editing, Resources, Project administration, Investigation, Funding acquisition, Formal analysis, Conceptualization.

## Declaration of competing interest

The authors declare that they have no known competing financial interests or personal relationships that could have appeared to influence the work reported in this paper.

## Figures and Tables

**Fig. S1:**
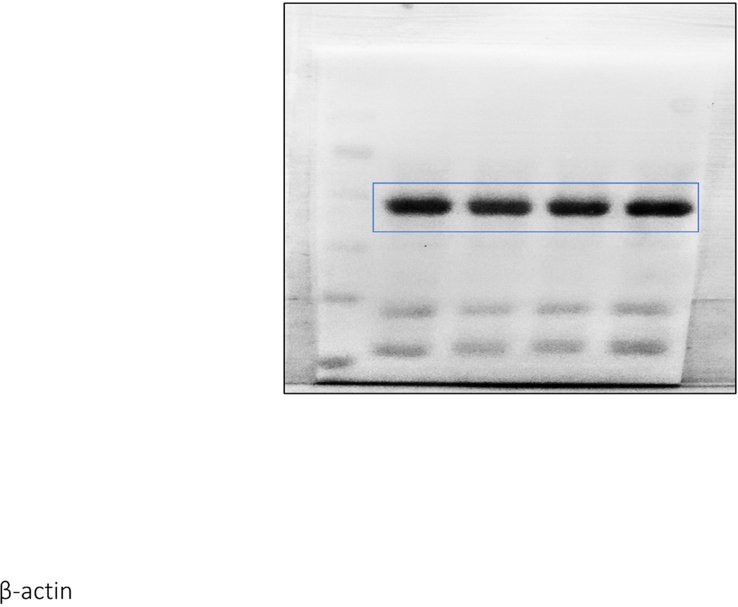


**Fig. S2:**
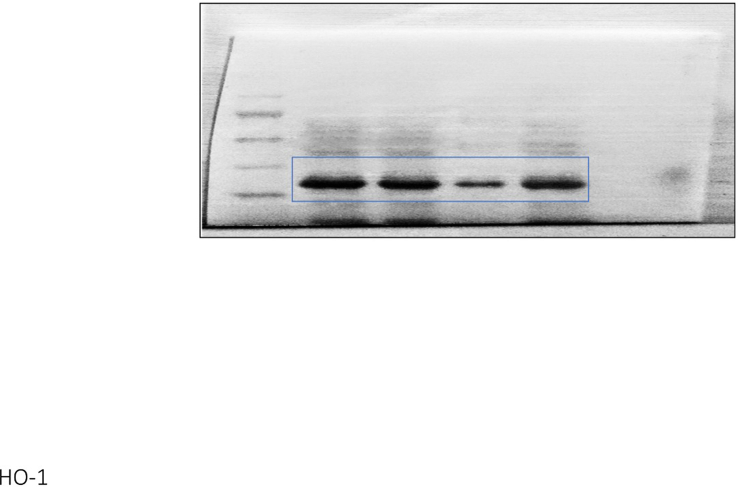


**Fig. S3:**
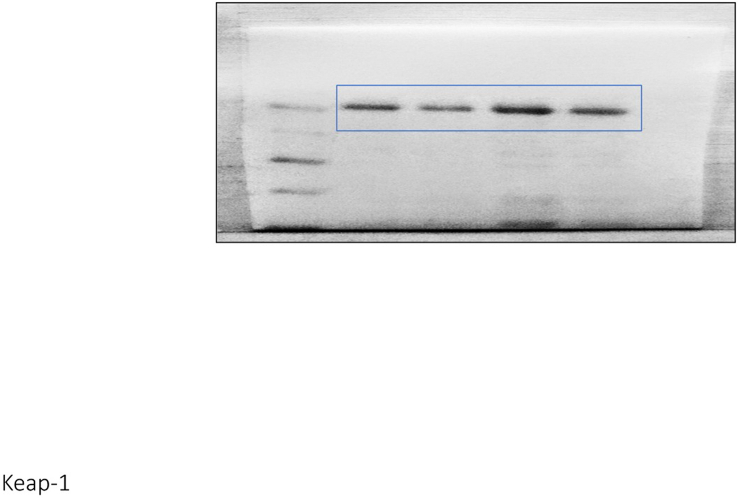


**Fig. S4:**
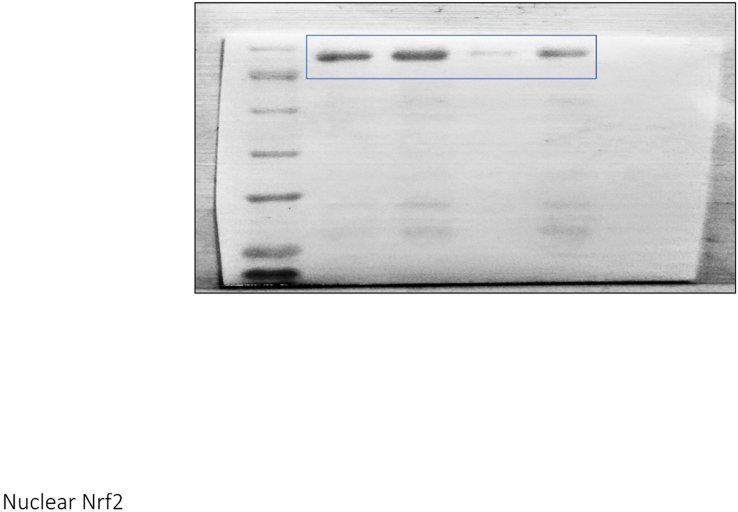


**figs5:**